# *Drosophila *as a genetic and cellular model for studies on axonal growth

**DOI:** 10.1186/1749-8104-2-9

**Published:** 2007-05-02

**Authors:** Natalia Sánchez-Soriano, Guy Tear, Paul Whitington, Andreas Prokop

**Affiliations:** 1 The Wellcome Trust Centre for Cell-Matrix Research, Faculty of Life Sciences, The University of Manchester, Manchester, UK; 2 MRC Centre for Developmental Neurobiology, Guy's Campus, King's College, London, UK; 3 Department of Anatomy and Cell Biology, University of Melbourne, Victoria, Australia

## Abstract

One of the most fascinating processes during nervous system development is the establishment of stereotypic neuronal networks. An essential step in this process is the outgrowth and precise navigation (pathfinding) of axons and dendrites towards their synaptic partner cells. This phenomenon was first described more than a century ago and, over the past decades, increasing insights have been gained into the cellular and molecular mechanisms regulating neuronal growth and navigation. Progress in this area has been greatly assisted by the use of simple and genetically tractable invertebrate model systems, such as the fruit fly *Drosophila melanogaster*. This review is dedicated to *Drosophila *as a genetic and cellular model to study axonal growth and demonstrates how it can and has been used for this research. We describe the various cellular systems of *Drosophila *used for such studies, insights into axonal growth cones and their cytoskeletal dynamics, and summarise identified molecular signalling pathways required for growth cone navigation, with particular focus on pathfinding decisions in the ventral nerve cord of *Drosophila *embryos. These *Drosophila*-specific aspects are viewed in the general context of our current knowledge about neuronal growth.

## Background

The function of a nervous system depends on the proper arrangement of its cellular elements, that is, neurons and glia cells. Amongst these, neurons bear axonal processes that establish synaptic contacts with other cells (neurons, muscles or gland cells) that can be a significant distance away. The transfer of information between these cells is the key feature of nervous system function and is usually mediated by action potentials that propagate along axons and are passed on to other cells at synapses. The wiring of such a system has to be precise and reproducible from individual to individual, as was first highlighted by Ramón y Cajal for the nervous systems of humans, other vertebrates, and also invertebrates [1]. Such precision is achieved during development through the guided growth of axons along specific paths, a process clearly governed by genetic mechanisms [2,3].

Essential work contributing to our current understanding of axonal growth has been carried out in vertebrates and invertebrates alike, in many instances demonstrating the conservation of principal mechanisms across the animal kingdom. One strategy towards improving molecular insights into axonal growth is the use of genetically tractable invertebrate model organisms, such as the worm *Caenorhabditis elegans *or the fruit fly *Drosophila melanogaster*. The strength of these species lies in their amenability to genetic manipulation (see below) and the fact that their nervous systems are composed of relatively low numbers of cellular elements. Whereas specific neuronal connections in vertebrates are usually formed by larger groups of neurons that develop and act in parallel, these connections are mostly represented by unique, individually recognisable neurons in invertebrates. Studies capitalising on such identifiable neurons, for example in insects, have helped to unravel principles of neuronal circuit formation. For example, the initial observation of guidepost cells as stepping stones for axonal growth came from studies of the grasshopper limb bud [4]. The concept of pioneer guidance, which proposes that axonal tracts are established by single pioneer neurons that are subsequently used as guidance cues for follower neurons, was discovered in vertebrates [5]. However, work on insects has helped to refine these concepts ('selective fasciculation' and 'labelled pathways' hypotheses) [6-8] and to contribute molecular players [9].

Once it was revealed that *Drosophila *embryos were experimentally accessible and largely homologous to grasshoppers at the single cell level [10], insights that had to that date mostly been gained on larger insects could be transferred into a genetically tractable model system. Such work in *Drosophila *was further fuelled by the development of new technologies, strategies and molecular tools, such as specific anatomical antibody probes [11,12] and the development of ever more sophisticated genetic screening strategies [13]. Furthermore, such research has profited enormously from the increasingly well organised research environment of *Drosophila*, including the systematic generation and provision at a large scale of new genetic tools and mutant fly stocks, and the improvement of access to research-relevant information [14-18].

Undoubtedly, the use of *Drosophila *as a model system for the study of axonal growth has been a prolific endeavour, providing a plethora of novel and/or refined insights into relevant molecular mechanisms, many of which have been shown to be conserved in higher organisms. This review will provide an overview of the major cellular model systems established for the fly, our current insights into *Drosophila *growth cones (the key structure executing axonal growth), the housekeeping machinery regulating the cytoskeletal dynamics required for axonal growth, and the signalling events involved in axon guidance, focussing on cellular models in the *Drosophila *embryo.

## Models for axonal growth in *Drosophila*

**Figure 1:**
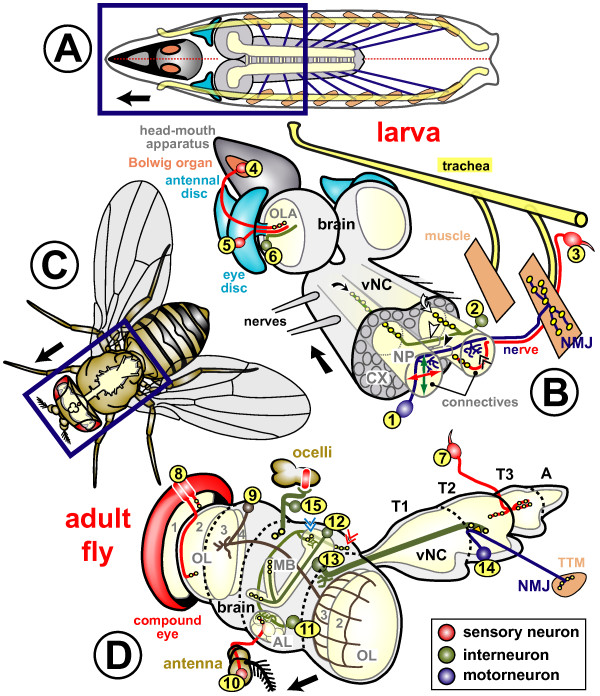
Neurons used for studies on neuronal growth at different stages of *Drosophila *development. **(a,c) **Horizontal views of a *Drosophila *larva and adult fly, respectively, illustrating the position of the CNS (grey and cream) in relation to other body structures. **(b,d) **Three-dimensional extracts from the areas boxed in dark blue in (a,c), respectively. The cell body area of the CNS (cortex (CX)) is shown in light grey, and the neuritic/synaptic area (neuropile (NP)) in cream (only relevant neuropile structures are shown in (b,d)). Black arrows point anterior, morphological structures are annotated in colour code, and neuronal classes are explained in the box at bottom right. The various model neurons are marked with numbers in yellow circles, explained below. Many neurons of the larval trunk can be studied from their birth in the embryo through to the mature synaptic stage. Amongst these, motorneurons (1) project towards the dorsal zone of ipsilateral or ipsi- and contralateral connectives (where they form dendrites; double chevron), from where they enter specific branches of peripheral nerves leading towards their target muscles, on which they form neuromuscular junctions (NMJ; yellow circles represent chemical synapses). Projections of larval interneurons (2) are restricted to the neuropile. Sensory neurons of the trunk (3) project along tracheal branches and motoraxons towards the ventral nerve cord (vNC) where they innervate the ventral domain of connectives [192,195,196,198-200,236]. Sensory neurons in the embryonic trunk have been used, for example, to study the actin-microtubule linker molecule Short stop, signalling through Robo or Notch receptors, or the spatial arrangement of axons in the neuropile [197,202,203,255]. Projections of neurons 1, 2 and 3 in the neuropile of the ventral nerve cord can be classified with respect to their anteroposterior extension within the segment (white curved arrow) or across segments (black curved arrow), their dorsoventral and mediolateral position in connectives (green and red double arrows, respectively), their ipsilateral (neuron 3) versus contralateral (neurons 1 and 2) nature, and their projection through anterior (white arrowhead) versus posterior commissure (black arrowhead; see details in 'Signalling mechanisms involved in axonal pathfinding in *Drosophila*' above). In the embryonic/larval head region (4), the Bolwig organ has been used for studies of neuronal growth. It contains somata of 12 photoreceptor cells [306], the axons of which form the Bolwig nerve projecting over the antennal and eye discs via the optic stalk into the optic lobe anlage (OLA) [26,307]. The Bolwig nerve is joined by successively outgrowing waves of axons of photoreceptor neurons (5), which are specified in the eye disc during larval and pupal stages. The optic lobe pioneer neuron (6), a projection neuron of embryonic origin, seems to be used as a guide within the OLA by the Bolwig nerve and photoreceptor axons [308,309]. Sensory neurons of the adult trunk (7) develop *de novo *during larval and pupal stages (with a few exceptions) [310] and terminate in the vNC neuropile (T1-3 and A indicate the three thoracic and fused abdominal segments). They can be analysed from the time of birth through to the fully differentiated stage [311,312], and have been used to study features, such as segment-specific growth regulation (homeotic genes), or the influence of adhesive interactions (*Dscam*), axonal transport (*cut up*, the dynein light chain) or of size alterations (*gigas*) on neuronal growth behaviour [311,313-315]. Photoreceptor cells in the adult compound eye (8) form a precise retinotopic map in the optic lobe (OL: grey 1, lamina; 2, medulla; 3, lobula; 4, lobula plate) established during larval (see neuron 5) and pupal stages, and the genetic mechanisms regulating these precise growth decisions are beginning to be unravelled [316-318]. Interneurons postsynaptic to photoreceptor neurons are well described [317,319] but seem not to have been used for studies of growth mechanisms so far, with the exception of a group of 20–30 dorsal cluster neurons (9; targeted by *atoGal4-14A*), which form dendrites in the ipsilateral optic lobe and project through the dorsal commissure to innervate the contralateral lobula and medulla [320-322]. Olfactory neurons in the third antennal segment (10) and the maxillary palp (not shown) project from the antenna into the antennal lobe (AL) where they terminate in specific glomeruli in a reproducible pattern correlating with the class of odorant receptor they express; the genetic regulation of this growth behaviour is under investigation [39]. The major output from the AL is constituted by projection neurons (11), which are postsynaptic to olfactory neurons and innervate the lateral horn (red double chevron) and the calyx (blue double chevron), a dorsal structure of the mushroom bodies (MB) [39]. The mushroom bodies are the brain structures responsible for olfactory learning in *Drosophila *[323,324], and its intrinsic interneurons (Kenyon cells (12)) project through the calyx and pedunculus where many of them bifurcate to project into the vertical α/α'-and the horizontal β/β'/γ-lobes, simultaneously [325]. The large giant fibre neuron (13) connects the optic system via a large diameter axon with motorneurons in the second thoracic segment (14), innervating the tergotrochanteral muscle (TTM; responsible for the visually induced jump escape response) via chemical and electrical (orange triangle) synapses [326]. Giant fibre axons grow out during late larval/pupal stages and have been used to study growth regulatory mechanism, such as the influence of Rho-like GTPases or the role of the E2 ubiquitin ligase Bendless [326]. Ocellar photoreceptor neurons do not send out their own axons but are connected to the brain via large interneurons, the cell bodies of which are located in the brain originally, but migrate into the periphery during pupal development (15). The pathfinding of these interneurons depends on a set of short-lived pioneer neurons that, in turn, require the extracellular matrix molecule laminin, the transmembrane receptor neurotactin and its ligand Amalgam for proper outgrowth [21,239,241]. Further potentially attractive models for studies of neuronal growth that are not shown here are auditory sensory neurons [327], and axonal fascicles in the ventral nerve cord of late *Drosophila *larvae representing paused interneurons of the future adult CNS (not shown) [209].

One of the major strengths of *Drosophila *lies in its use as a model where axonal growth can be studied *in situ*. A widely used strategy for the unbiased discovery of neuronal growth mechanisms in *Drosophila *is the search for mutations that cause morphological aberrations of axonal tracts or neuronal connections *in situ*. Subsequently, the genes associated with such mutations can be identified, and the molecular nature, function and type of interactions of their products can be studied. To facilitate such genetic screens and functional studies of gene action, descriptions of axonal pathways and neuronal connections in wild-type animals have been provided for a number of different neural systems in *Drosophila*. A selection of these cellular models is illustrated in Figure 1, and some of their principal features will be discussed below.

**Figure 2:**
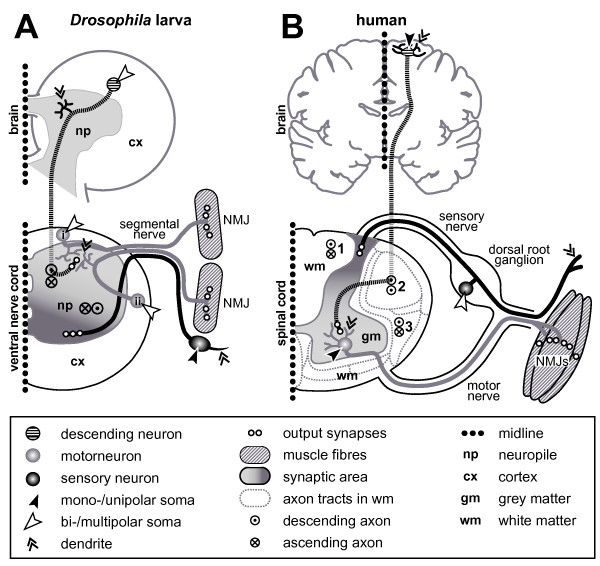
Comparing principal features of neuronal organisation and growth in *Drosophila *(larva) and vertebrates (human). **(a) **Saggital section (one body half; dotted line is midline) through the larval brain and ventral nerve cord (compare Figure 1a,b). **(b) **Saggital section through the adult human brain and one half of the spinal cord. Symbols are explained in the box below. Whereas axons of unipolar inter- and motorneurons in *Drosophila *have to grow into the synaptic area where they form dendrites, comparable neurons in vertebrates are multipolar and locate themselves in the synaptic area. All *Drosophila *motorneurons locate their dendrites in the dorsal neuropile, regardless of their soma position (see 'i' versus 'ii') [236]. *Vice versa*, sensory somata in *Drosophila *are located next to their dendrites, whereas cell bodies of most sensory neurons in vertebrates are grouped together in the dorsal root ganglia. Sensory output (dark grey) and motor input areas (bright grey) are inverted in both phyla, which might be explained through a general dorsoventral body axis inversion between vertebrates and arthropods [328], that is, not represent an organisational difference between their CNS. Ascending/descending axons in *Drosophila *are non-myelinated and project through the synaptic area (compare neurons 2 and 13 in Figure 1) where they take on characteristic positions [236,329]. In vertebrates, ascending/descending axons are myelinated and positioned outside the synaptic area, grouping into characteristic tracts in defined positions of the white matter; examples named here: *fasciculus cuneatus *(1), *tractus corticospinalis lateralis *(2; pyramidal tract; only descending), and *tractus spinothalamicus lateralis *(3).

First, neurons of all classes, that is, sensory neurons, interneurons and motorneurons, have been used for studies of axonal growth in *Drosophila *(red, green and blue in Figure 1, respectively). The principal structures of vertebrate and invertebrate neurons have been proposed to be homologous [19], despite there being certain organisational differences (Figure 2). These differences may be associated with deviations in some aspects of axonal pathfinding behaviours (details in Figure 2). For example, in the vertebrate trunk, sensory neurons are (pseudo-)unipolar and located in the dorsal root ganglia derived from a migratory stream of neural crest cells [20]. In contrast, cell bodies of sensory neurons in *Drosophila *are usually bi- or multipolar and are born and located in the periphery close to the sense organs they innervate or represent. Therefore, sensory axons in *Drosophila *grow unidirectionally towards the central nervous system (CNS), whereas sensory neurites in vertebrates bifurcate and grow bidirectionally both towards the CNS and into the periphery. A clear exception to this rule are the ocellar photoreceptors of *Drosophila *(located on the dorsal surface of the head; Figure 1c,d), which do not form axons themselves, but are connected to the brain via interneurons [21]. Somata of inter- and motorneurons are commonly multipolar in vertebrates and located in the synaptic region (grey matter; Figure 2b). In contrast, cell bodies of comparable neurons in *Drosophila *are unipolar and localised outside the neuropile within the cortex of the neuromeres. Finally, in vertebrates, axons ascending and descending to/from the brain are located in defined tracts in the white matter (1–3 in Figure 2b) where they become heavily myelinated, whereas comparable axons in *Drosophila *are located in the synaptic neuropile and usually lack glial ensheathment. Hence, mechanisms placing ascending/descending axons in the *Drosophila *neuropile might be distinct from those placing them in the white matter in vertebrates. Alternatively, if the same guidance cues are utilised, their spatial expression must differ considerably between vertebrates and arthropods.

Second, *Drosophila *is a holometabolous insect that exists as a maggot during larval life but is substantially reorganised into the adult fly during metamorphosis at the intermediate pupal stage. This process is not dissimilar to metamorphic events known from lower vertebrates, such as amphibians. One phase of *de novo *axonal growth takes place in the embryo, and a vast number of these cellular elements are maintained into the adult stage, albeit being remodelled during the pupal period [22]. However, to adapt to the far more complex behavioural repertoires of the adult, a second phase of *de novo *neurogenesis and axonal growth is initiated during larval life and completed during the process of metamorphosis. There is abundant evidence that common cellular and molecular mechanisms for axon guidance apply at both stages, allowing us, in many instances, to study the same molecules with the multiple techniques and cellular systems available for both stages of axonal growth in *Drosophila *[3].

Cellular models from both developmental phases (Figure 1a,b versus 1c,d) have been used to screen for genes involved in neuronal growth regulation. At embryonic/larval stages, genetic screens have mostly been based on analyses of animals bearing mutations that were induced by chemical treatment, radiation or via transposable elements. Morphological read-outs used in these screens have included motornerves, projections in the CNS, or sensory projections in the trunk or head (for example, Bolwig's organ; 1–4 in Figure 1) [23-32]. Some screens have been carried out on adult flies using as read-outs the giant fibre system (13 and 14 in Figure 1) or general brain morphology [33,34]. However, since many mutations cause embryonic or larval lethality, the utility of adult animals for genetic screens is limited. To overcome this problem, mosaic strategies have been used in which only specific tissues express the mutant phenotype and can be maintained by an otherwise normal body [13]. Such mosaic screens have been successfully carried out using adult photoreceptor axons as read-outs (8 in Figure 1) [35,36]. A further refined mosaic strategy is the MARCM technique, in which homozygous mutant neurons can be visualised at the single cell level, surrounded by heterozygous, non-mutant cells [37]. This technique has been used to study and screen for growth aberrations of mushroom body, olfactory and photoreceptor neurons (8, 10 and 12 in Figure 1) [38-40]. Based on these various screens in embryonic, larval or adult individuals, many mutations that result in defective axon growth have been identified [3]. The gene functions underlying a number of these mutations will be discussed below (see 'Regulators of actin dynamics in *Drosophila *growth cones' below).

Studies of neurons *in situ *have the essential advantage that they address the relevance of genetic mechanisms for developmental processes in a natural context. However, the ability to utilise precise and well-controlled pharmacological, physiological or imaging methods in such *in situ *systems is limited. To address these shortcomings, cultures of primary neurons, obtained from embryonic or larval tissues, have been established in *Drosophila*. These primary culture systems have been used successfully to demonstrate and/or analyse various phenomena, such as promotion of axonal growth by the neural cell adhesion molecule (N-CAM) homologue Fasciclin 2 or the extracellular matrix molecule laminin [41-43], induction of axonal fasciculation through neuronal activity [44], dependence of axonal growth on endocytosis [45,46], or cAMP-dependent neurotransmitter release from growing neurons [47]. All of these phenomena are likely to be of relevance to neuronal growth *in situ *[9,21,48], and the established culture systems provide valuable additional means for their study.

Taken together, the *Drosophila *system provides a wide range of cellular models to be used for studies of axonal growth. In addition, a number of models exist for other forms of neuronal growth, such as dendritic arborisation or the branching and plasticity of synaptic terminals [49-52]. However, these systems will not be considered here.

## Growth cone dynamics in *Drosophila*

### Principal structure and function of growth cones

The elongation of an axon is led at its tip by a highly dynamic structure, the growth cone, first described and named by S Ramón y Cajal ("*cono de crecimiento*") and then confirmed in live studies by RG Harrison [53-55]. Growth cones navigate along stereotypical paths, steadily elongating the axon by adding new structural components such as microtubules and membrane to its tip [56]. Just like the leading front of migrating cells, growth cones display actomyosin-containing filopodia and lamellipodia, focal contacts, a dynamic population of microtubules, and cell polarity markers such as Par-3 and -6 [57-62]. In contrast to migrating cells, where stress fibres pull the entire cell forward, neuronal cell bodies generally remain behind when growth cones advance, remaining connected by the steadily elongating axons, the core of which contains bundled, stable microtubules representing the highways for axonal transport.

It has been shown for growth cones of many species, that their guidance requires actin dynamics in the growth cone periphery [55,59,60,63]. The peripheral filamentous actin cytoskeleton executes a continuous, myosin-driven retrograde flow that involves ATP-dependent addition of globular actin to actin filaments at their barbed ends (pointing towards the growth cone's periphery), a gradual change of actin-bound ATP to ADP, and hydrolysis or severing at their pointed ends (pointing towards the growth cone's centre) [64-66]. This process of actin assembly and disassembly and its organisation into lamellipodial networks and filopodial bundles is regulated by a complex molecular machinery [65,67-69], which we detail below in the context of *Drosophila *components (see 'Regulators of actin dynamics in *Drosophila *growth cones').

Whereas growth cone guidance crucially depends on actin dynamics, growth cone advance depends on microtubules. This is clearly demonstrated by the persistence of axonal growth in the presence of actin polymerisation blockers, in vertebrates as well as in *Drosophila *[55,70-72], whereas pharmacological destabilisation of microtubules causes growth cone retraction [55,73]. Furthermore, extending axons can even be induced to turn in the absence of filamentous actin if they are exposed to an electrical field [74]. Microtubules in the axon shaft and central zone of growth cones are stable and bundled, whereas single unstable microtubules elongate into and retract from the peripheral actin-rich zone in a highly dynamic fashion. These microtubules can be trapped or stabilised through signals in the growth cone periphery, thus determining the direction in which microtubules of the axon shaft will extend [55,58-60,63]. Microtubule growth and shrinkage is, *per se*, an autocatalytic process [75-77]. However, a number of cellular factors regulate microtubule dynamics, as detailed below in the context of *Drosophila *components (see 'Regulators of microtubule dynamics in *Drosophila *growth cones').

### Growth cones in *Drosophila*

Initial work on *Drosophila *growth cones dates back more than 20 years when growth cones were first described incidentally in a study on the role of the activity regulator Maleless/Nap in primary neurons cultured from the larval nervous systems [78]. *In vivo *observations carried out on growth cones of primary neurons in culture demonstrated classifiable shape differences (longer filopodia of ventral ganglion-derived neurons versus those derived from brain), and also established strategies to measure their dynamics [79]. These strategies were later used to demonstrate that genetic or pharmacological alterations of cAMP levels influence growth cone dynamics [80]. Analyses of dye-filled motorneurons in *Drosophila *embryos demonstrated that their growth cones make characteristic and reproducible directional decisions when advancing in the CNS and periphery [81]. In agreement with this finding, ultrastructural studies in the developing embryonic *Drosophila *CNS showed that filopodia of pioneering growth cones establish close contacts with characteristic sets of cells in their immediate environment [82]. *In vivo *studies of the identified RP2 motorneuron in the same context revealed that these growth cones grow at rates comparable to other animal model systems and carry out a set of characteristic shape changes along their reproducible paths [83]. Follow-up studies using this system showed that genetic removal of the Roundabout receptor (see 'Mediolateral patterning of longitudinal fascicles' and 'Growth cone guidance at the *Drosophila *CNS midline' sections below) in these growth cones led to an increase in the length and persistence of filopodia [84]. *In vivo *observations on growth cones of a different set of motorneurons (ISNb) demonstrated that their filopodial dynamics and guidance are regulated through GTPases of the Rho-family and their downstream effectors [85,86]. Further studies on embryonic motorneurons have shown that growth cones of pioneer neurons display a significantly more complex shape than their follower axons [9], a finding that is consistent with descriptions of the growth cones of pioneer/follower neurons in vertebrates [87,88]. In the *Drosophila *system, this behaviour is regulated by the N-CAM homologue Fasciclin 2 [9]. Motorneuronal growth cones have also been studied in their transition phase into a characteristically shaped neuromuscular junction. Such analyses have revealed how growth cones, which initially explore a wider muscle field, transform into precisely targeted neuromuscular terminals [89,90]. Ultrastructural analyses of such growth cones have shown how filopodia of identified motorneurons establish intimate contacts only with their specific target muscles, and that this process is regulated through the presentation of specific cell recognition molecules [91]. Consequently, ablation of such target muscles changes the behaviour of growth cones in their target area [92].

**Figure 3:**
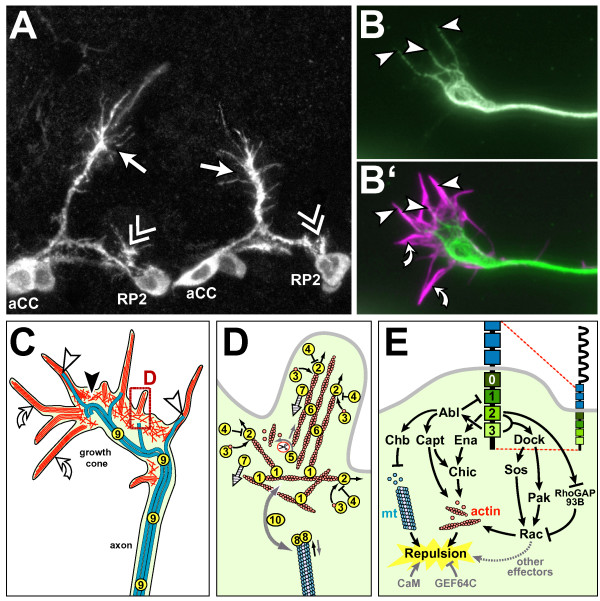
*Drosophila *growth cones and the (potential) factors regulating their cytoskeletal dynamics. **(a) **Growth cones of aCC (arrows) and RP2 motorneurons (double chevrons; cell bodies named) in two consecutive segments of the trunk of a *Drosophila *embryo, stained with a cell-specifically expressed membrane marker. **(b,b') **Cultured *Drosophila *growth cone stained for microtubules (green) and filamentous actin (magenta); some filopodia lack microtubules (curved arrows), whereas others are deeply invaded (arrow heads indicate microtubule tips). **(c) **Schematic representation of the cytoskeletal organisation in *Drosophila *growth cones as extrapolated from work on growth cones in other species (detailed in the section 'Principal structure and function of growth cones'): veil-like lamellipodia (black arrowhead) contain mesh-like networks of actin filaments (randomly oriented red lines), whereas pointed filopodia (white arrowhead) contain bundled actin filaments (parallel red lines); microtubules (blue lines) are bundled in the axon, but single splayed microtubules extend into the periphery of the growth cone (curved white arrows indicate splayed microtubule tips), reaching into filopodia, as was similarly reported for growth cones of other species or migrating cells [63,330]. **(d) **Details of the boxed area in (c); circled numbers correlate with the numbers in Table 1 and represent the following molecular activities: 1, actin filament nucleation by Arp2/3 (which subsequently stays with the pointed ends); 2, actin filament nucleation and elongation by formins (which stay with barbed ends); 3, actin monomer binding; 4, barbed-end capping; 5, pointed end-depolymerisation/severing; 6, actin filament bundling; 7, retrograde flow of actin cytoskeleton; 8, microtubule plus end binding; 9, microtubule stabilising; 10, actin-microtubule linkage. Black straight arrows indicate growth of actin filaments or microtubules, grey straight arrows shrinkage, black curved arrows addition of actin monomers, grey curved arrows removal of actin monomers or filamentous fragments, hatched arrows indicate direction of retrograde actin flow, and the grey dashed curved double arrow linkage of actin and microtubules. **(e) **Current view of the effectors downstream of the Slit receptor Robo mediating repulsion from the midline of the ventral nerve cord. Robo (top right) habours five immunoglobulin domains (half elipses) and three fibronectin type III domains (blue boxes) extracellularly, and four conserved cytoplasmic (CC) domains (light to dark green) intracellularly. Robo induces growth cone repulsion by controlling cytoskeletal dynamics via either Abelson kinase (Abl) and Enabled (Ena), or Rac activity. Ena binds at CC2 and acts most likely through Chickadee/Profilin on actin dynamics. Abl binding to Robo at CC3 influences actin dynamics via Capulet and microtubule dynamics via the +TIP protein Chromosome Bows (Chb/Orbit/MAST). Simultaneously, Abl phosphorylates CC1 to antagonise Robo function. The regulation of Rac activity through Robo occurs through CC2/3 recruitment of the SH3-SH2 adaptor molecule Dreadlocks (Dock) which, in turn, activates Rac through both Pak and the GEF Sos. In parallel, active Robo can influence Rac activity via the binding of RhoGAP93B (vilse/CrGAP) to CC2, but it remains unclear whether RhoGAP93B is positively or negatively regulated by Robo. Paradoxically, both decrease and increase of Rac activation levels can cause midline crossing, suggesting that: Rac might influence other effectors to cause repulsion; a precise Rac activation level is required to mediate Slit-induced repulsion; or a sequential modification of Rac in response to Robo activation has to occur, such as an initial role to prevent extension towards the source of the repellent and another role to encourage extension away from the Slit source. Calmodulin and GEF64C have additionally been identified as modifiers of Robo activity, although it is not clear yet how they influence Robo signalling (calmodulin possibly through Chic).

*Drosophila *growth cones display the characteristic hallmarks of those in other systems, such as highly dynamic filopodia, enrichment of microtubules at their core and the presence of filamentous actin in their periphery (Figure 3). Yet, to date, subcellular studies of *Drosophila *growth cones are fairly sparse. Most of the insights into axonal growth mechanisms in *Drosophila *(as reviewed below) have been derived from analyses of the axonal projection defects found in the mature embryonic or adult nervous system in mutant animals or under experimentally manipulated conditions. There are only few descriptions of the behaviours of growth cones during the axon elongation phase in these animals.

### Regulators of actin dynamics in *Drosophila *growth cones

The morphological studies of *Drosophila *growth cones described above ('Growth cones in *Drosophila*' section) suggest that they possess the same cytoskeletal machinery as found in other model organisms ('Principal structure and function of growth cones' section). In this section we summarise and speculate on the (potential) factors regulating cytoskeletal dynamics in *Drosophila *growth cones. An overview of these components is given in Figure 3 and Table 1.

**Table 1:** Direct regulators of the cytoskeleton with (potential) functions at growth cones

Gene name	Common synonyms	Direct regulators	References
**Actin filament nucleation and elongation (1 + 2)**			
*Suppressor of profilin 2 *(*Sop2*)	*ArpC1*	*WASp *(+), SCAR/WAVE complex (+; containing: *SCAR*/WAVE, *Hem*/*kette*, *Sra-1*/*CYFIP*, *Abi*, *SIP1*/*HSPC300*)	[95,149]
*Actin-related protein 66B *(*Arp66B*)	Arp3		
*Arp14D*, *Arc-p34*, *Arpc3A/3B*, *Arc-p20*, *p16-Arc*	Arp2, ArpC2-C5		[93,94]
*dishevelled associated activator of morphogenesis *(*DAAM*)	NA	?	*
*enabled *(*ena*)	*ENA/VASP*	*Abelson *(-), Dlar (+)	[100,101,164]
			
**Actin monomer binding (3)**			
*capulet *(*capt*)	*acu*, *CAP*	Abelson (+)	[103]
*ciboulot *(*cib*)	NA	?	[102]
*chickadee *(*chic*)	*profilin*, *sand*	ena (+)	[101,164]
*twinfilin (twf)*	NA	?	[104]
			
**Barbed-end capping (4)**			
*capping protein α *(*cpa*)	NA	?	[109]
*capping protein β *(*cpb*)	NA	?	[333]
			
**Pointed-end depolymerisation/severing (5)**			
*twinstar *(*tsr*)	*ADF/cofilin*	*LIMK1 *(-), *ssh *(+)	[111]
*flightless I *(*fliI*)	*(gelsolin family)*	?	[115]
*quail *(*qua*)	*villin-related (gelsolin family)*	?	[116]
			
**Actin filament bundling (6)**			
*singed *(*sn*)	*fascin*	?	[126]
*α Actinin *(*Actn*)	*flightless A*	?	[128]
*Fimbrin *(*Fim*)	NA	?	[334]
*cheerio *(*cher*)	*filamin*	?	[129]
			
**Retrograde flow of filamentous actin (7)**			
*zipper *(*zip*)	*myosin heavy chain*	?	[121]
*spaghetti squash *(*sqh*)	*myosin II light chain*	*Strn-Mlck *(+), *Mbs*/*myosin phosphatase *(-)	[119,120]
			
**Microtubule plus end binding (+TIPs) (8)**			
*CLIP-190*	NA	?	[152]
*chromosome bows *(*chb*)	*CLASP*, *Orbit/MAST*	*Abelson *(+)	[153]
*eb1*, *CG18190*, *CG32371*	NA	?	[155]
*APC-like *(*Apc*)	*Apc1*	?	[154]
*Apc2*	NA	?	[154]
			
**Microtubule stabilising (9)**			
*futsch*	*MAP1B*, mAB22C10	*Fmr1*/*fragileX*/*fmrp *(-)	[163,335]
*tau*	NA	?	[161]
			
**Microtubule-actin linkage (10)**			
*short stop *(*shot*)	*kakapo*, *groovin*	?	[168]
*pod-1*	NA	?	[169]

The table lists official gene names according to FlyBase [14] (first column), examples of commonly used synonyms (second column; NA, not applicable), molecules reported to regulate the respective gene/protein directly (third column; '+', activating; '-', down-regulating), and representative references (fourth column; for more information refer to FlyBase; preferentially those references are listed that link to the nervous system). Bold headings indicate a functional assignment of the factors listed below, and numbers refer directly to the circled numbers in Figure 3. *See section 'Regulators of actin dynamics in *Drosophila *growth cones' for more detail.

**Figure 4:**
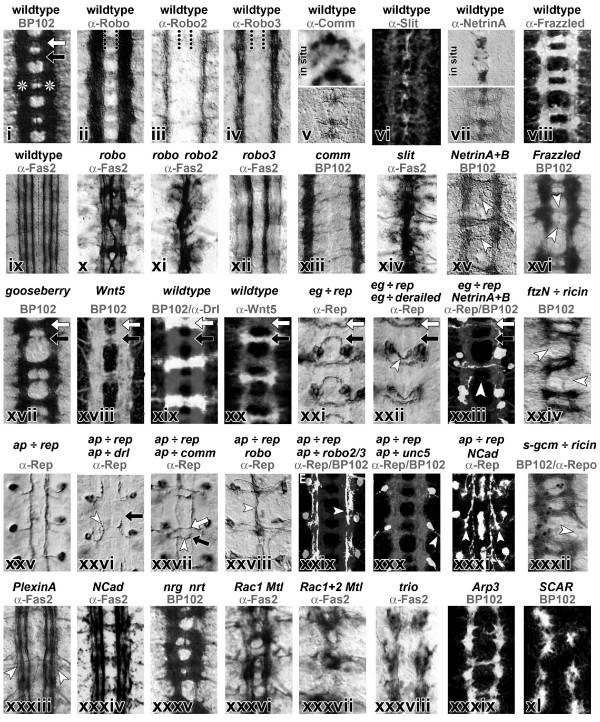
Representative embryonic mutant phenotypes of axonal projections in the ventral nerve cord. Images of ventral nerve cords in horizontal view (dorsal up) of embryos that are wild type, mutant or display targeted expression of genes in subsets of neurons. Genotypes are indicated in black at the top, antibody stainings in grey (abbreviations: Robo, Roundabout; Comm, Commissureless; Fas2, Fasciclin 2; Drl, Derailed; Rep, reporter gene; *eg*, *eagle*; *ftz*, *fushi tarazu*; *ap*, *apterous*; *unc5*, *uncoordinated 5*; *NCad*, N-Cadherin; *gcm*, *glia cells missing*; *nrg*, *neuroglian*; *nrt*, *neurotactin; *"X ÷ Y", expression of gene Y driven by the promoter of gene X). BP102 antiserum labels the complete neuropile (i), consisting of two connectives (asterisks) and, per segment, an anterior (white arrow) and posterior (black arrow) commissure. At stage 16 Fasciclin 2 labels three prominent longitudinal fascicles per connective (ix). (ii-viii) Expression patterns of genes involved in midline crossing and, below (x-xvi), respective loss-of-function phenotypes (see text for details). (xvii-xxii) Regulation of growth through the anterior versus posterior commissure, with loss of posterior (xvii) or anterior (xviii) commissure, specific expression of Derailed (xix) in anterior and its ligand Wnt (xx) in posterior commissure, and shift of posterior commissure neurons (*eagle-Gal4*; xxi) to the anterior commissure (white arrow head in xxii) upon Derailed expression in their axons. (xxiii) Same eagle-Gal4 neurons partially lack commissural projections in netrin A*+B *mutant background (compare white arrowhead in (xv)). (xxv-xxxi) Detailed phenotypic studies using identified *apterous-Gal4 *neurons, which project transversely to the medial connectives where they form a longitudinal fascicle (xxv); as indicated by white arrows, they stall prematurely upon Derailed expression (xxvi), project across the midline upon Comm expression (xxvii), collapse towards the midline in *robo *mutant background (compare x), shift to lateral positions upon Robo2+3 expression (xxix), project out of the CNS upon Unc5 expression (xxx), or turn prematurely from transverse into longitudinal direction in *N-cadherin *mutant background (xxxi). (xxiv, xxxii). Connectives are affected upon ablation of longitudinal pioneer neurons (xxiv) or longitudinal glia cells (xxxii). (xxxiii-xl) Various neuropile phenotypes in embryos mutant for transmembrane molecules (xxxiii-xxxv) or factors involved in cytoskeletal regulation (xxxvi-xl). Images were taken, with permission, from [241] (i, xxxv), [253] (ii-iv, xii, xxix), [207] (v, top), [331] (v, bottom), [259] (vi), [266] (vii, xv), [225] (viii), [244] (ix, xxxiii), [271] (x, xi, xiv), [23] (xiii, xvii), [212] (xvi, xxx), [263] (xviii), [208] (xix, xxi, xxii, xxv-xxvii), [332] (xx), [267] (xxiii), [219] (xxiv), [277] (xxviii), [226] (xxxi, xxxiv), [223] (xxxii), [132] (xxxvi-xxxviii), [95] (xxxix, xxxx). Images were modified to grayscale and adapted to size.

Several factors have been reported to be able to seed new actin filaments, a process called 'nucleation'. One of these factors is the Arp2/3 (actin-related protein) complex, which is composed of seven subunits. The genes encoding all its subunits have been identified in *Drosophila *and some of them have been functionally assessed [93,94]. Loss of function of the genes *Suppressor of profilin 2 *(ArpC1) and/or *Arp66B *(Arp3) causes severe axonal projection defects when analysed at late embryonic stages (Figure 4, xxxix) [95]. Another independent nucleator of actin filaments, Spire, has been described for *Drosophila *[96] but seems not to be localised in the nervous system [14]. As opposed to Arp2/3-complex or Spire, members of the Formin family of proteins are nucleators and, in addition, effective barbed end-binding elongators of actin filaments [97]. Of the six *Drosophila *Formins [98], DAAM (dishevelled associated activator of morphogenesis) is expressed pan-neurally while two others, *CG32030 *and *CG32138*, are specifically expressed within midline glia (the *AA142 *transposon insertion, a classic midline glia marker, is inserted within *CG32030*; Richard Tuxworth and GT, unpublished observations). The other Formins, *cappuccino*, *diaphanous *and *formin3*, do not appear to be expressed at detectable levels in developing neurons [98]. No neural phenotypes have yet been associated with mutations in either of these genes, but ectopic expression of Formin 3 in the CNS can induce strong pathfinding phenotypes [98]. Thus, based on our current knowledge, the Arp2/3 complex and DAAM are the potential actin nucleators in *Drosophila *growth cones. Of these, Arp2/3 is unlikely to regulate subsequent actin filament elongation, since it stays with the pointed ends of elongating actin filaments, which move away from the very cell periphery after nucleation has occurred [68,97,99]. Instead other factors have been proposed to facilitate actin filament elongation. Likely candidates in *Drosophila *are DAAM (see above) or Enabled, the *Drosophila *member of the Enabled/VASP (vasodilator-stimulated phosphoprotein) family of proteins, which is a direct target of the cytoplasmic Abelson tyrosine kinase in the context of neuronal growth [100,101]. Further factors regulating actin assembly are molecules that bind monomeric actin, such as Capulet, Ciboulot and Profilin/Chickadee, all of which are essential for neuronal growth regulation in *Drosophila *[101-103]. A further actin monomer-binding molecule, Twinfilin, is likely to be expressed in the nervous system, but no data exist concerning its cellular function [104].

Profilin/Chickadee has been shown to interact with Calmodulin [105], suggesting [Ca^2+^]_i _may influence its activity. Consistent with this observation, growth cones of *Drosophila*, like those of other species, show measurable changes in local calcium concentration [106-108]. Barbed-end capping proteins are typical inhibitors of actin polymerisation. For *Drosophila*, the capping proteins Cpa and Cpb have been reported, but their role in the nervous system has yet to be addressed [109].

While actin filaments elongate at their barbed ends, they are severed and/or depolymerised at their pointed ends, and a number of factors have been assigned to this process [110]. One of these factors is ADF/cofilin (Twinstar in *Drosophila*), the activity of which is directly regulated by LIM kinase and the Slingshot phosphatase in vertebrate growth cones and the developing *Drosophila *nervous system alike [111-113]. Another group of potent filamentous actin severing molecules comprises members of the gelsolin family [114], at least two of which have been identified in *Drosophila*, flightless I and quail [115,116]. However, nothing is known about their potential requirement for axonal growth.

Distal elongation and proximal shortening of actin filaments, generally referred to as 'treadmilling', would potentially lead to a constant outward movement of actin filaments. However, such movement is antagonised by myosin motor proteins, which produce a steady retrograde/reaward flow of the actin network. This action of myosin II plays an important regulatory role downstream of guidance-related signalling events [58,117,118]. Accordingly, two subunits of non-muscle myosin, the heavy chain (Zipper) and the regulatory light chain (Spaghetti-squash), and the direct regulators myosin light chain kinase (MLCK) and myosin light chain phosphatase have been implicated in neuronal growth in *Drosophila *[119-121].

Accumulating evidence suggests that the set of actin regulators involved in lamellipodia formation (producing branching networks of filamentous actin) is not congruent to those in filopodia (bundled linear actin filaments) [69]. The typical parallel bundling of actin filaments in filopodia can be initiated by the actin polymerisation factor Enabled/VASP in collaboration with Formins [122,123], and the principle involvement of both these factors in axonal growth of *Drosophila *has been reported (see above). Another suggested mode of filopodium formation is through bundling of filamentous actin via the powerful cross-linker Fascin [124,125]. Singed, the *Drosophila *orthologue of Fascin, has been shown to influence neuronal growth [126]. Other factors cross-linking actin filaments, such as α Actinin, Fimbrin or Filamin (*cheerio *in *Drosophila*) are able to substitute Fascin in the context of *Listeria *propulsion [127]. In *Drosophila*, *α Actinin *is expressed in the developing CNS [128], and *cheerio *has been associated with associative learning [129], but none of these factors has been studied in the context of neuronal growth so far [14].

The complex machinery mediating actin dynamics in growth cones is essentially orchestrated by Rho-family GTPases [130,131], six of which have been reported for *Drosophila*. Of these, Rac1, Rac2 and Mtl have an overlapping requirement during the regulation of axon extension, branching and guidance (Figure 4, xxxvi and xxxvii) [132,133]. Cdc42 is similarly involved in growth cone guidance, but its functions appear to involve downstream effectors different from those of Rac1 (for example, Pak) [85,86,134]. RhoA has been shown to inhibit neuronal growth in *Drosophila *[135], whereas little is known about the other two Rho-like GTPases, RhoL and RhoBTB [136]. Many of the factors shown to act up- or downstream of Rho-GTPases in vertebrates have been identified in *Drosophila *and their functions in neuronal growth were revealed by their mutant phenotypes. Thus, GTPases are active in a GTP-bound state, promoted by RhoGEFs (guanine nucleotide exchange factors), and inactive in a GDP-bound state, catalysed by RhoGAPs (GTPase activating proteins) [131]. The *Drosophila *genome contains 20 predicted RhoGAPs and 22 RhoGEFs [137-139]. At least three RhoGEFs, Still life, Gef64C and Trio, have been associated with neuronal growth regulation of the axons of motorneurons, photoreceptors, and neurons in the ventral nerve cord (Figure 4, xxxviii) [138,140-142]. Three of the 20 RhoGAPs, RhoGAP-71E1, RhoGAP-50C14 and RhoGAP-16B12/p190, show neuronal growth phenotypes when assayed in the mushroom body neuropile (MB in Figure 1d) [139], and RhoGAP93B/CrGAP/Vilse is involved in midline crossing of axons (see 'Growth cone guidance at the *Drosophila *CNS midline' below) [143,144]. An involvement in neuronal growth regulation has also been demonstrated in various cellular contexts for typical effectors of Rho-like GTPases, such as p21-activated kinase (PAK) and its closely associated partner, the SH2/SH3 adaptor protein Nck (Dreadlocks = Dock in *Drosophila*), the RhoA effector Rho-kinase (Drok or Rock in *Drosophila*), and the Cdc42 effector WASP, a direct activator of the Arp2/3 complex [95,141,145,146]. WASP's paralogue SCAR in itself is not an immediate effector of GTPases, but the WAVE/SCAR complex (containing SCAR, CYFIP, Kette, Abi and HSPC300) can be targeted by Rac through its direct interaction with CYFIP [147,148]. The WAVE/SCAR complex activates the Arp2/3 complex and has a clear impact on neuronal growth (Figure 4, xl) [95,149].

Taken together, most if not all classes of actin regulatory factors have been identified in *Drosophila *and many of them have been associated to varying degrees of certainty with neuronal growth.

### Regulators of microtubule dynamics in *Drosophila *growth cones

The second cytoskeletal component essential for growth cone advance is the microtubule; factors regulating their dynamics are summarised in Figure 3 and Table 1. One important class of proteins regulating the elongation and shortening of microtubules comprises the plus-end-tracking molecules (+TIPs) [150,151]. A number of these have been identified in *Drosophila*. CLIP-190 (orthologue of vertebrate CLIP-170) is strongly expressed in the nervous system [152] and has been shown to interact with the +TIP protein Chb (Chromosome bows/Orbit/MAST, an orthologue of vertebrate CLASP, CLIP-associated protein) [153]. Further +TIP molecules described for *Drosophila *are APC1 and APC2 (adenomatous polyposis coli), both of which are expressed in the brain (though mainly in neuronal somata) [154], and three possible homologues of EB1 (Endbinding protein 1; *Eb1*, *CG18190*, and *CG32371*), the potential role of which has yet to be addressed in the nervous system [155]. A potential regulator of these molecules is the Par3/6-complex, which localises to growth cones in both mammals and *Drosophila*, and has been shown to organise the cytoskeleton in *Drosophila *motorneurons [19,57,156,157]. Another molecule required for neuronal growth in *Drosophila *is the lissencephaly-associated factor Lis1, a molecule shown to be capable of binding microtubules to reduce microtubule catastrophe events [158,159]. Characterisation of the *lis1 *mutant growth phenotype in *Drosophila *suggests Lis1 may function through an interaction with the motor protein Dynein [158,160]. Finally, an important class of microtubule-binding proteins, also reported for *Drosophila*, comprises the microtubule associated proteins (MAPs), which stabilise and facilitate transport along microtubules [151]. MAP2 seems to be absent from the *Drosophila *genome, but one *Drosophila *Tau-like protein that localises within axons has been described, although no functional data have been reported to date [161,162]. A MAP1B-like molecule called Futsch has been shown to regulate axonal growth in *Drosophila*, presumably through stabilisation and loop formation of microtubules [163].

Therefore, as for the actin cytoskeleton, many regulators of microtubule dynamics in the *Drosophila *nervous system have been identified. Interestingly, the proteins described here are regulated in part through the same factors described previously in relation to actin dynamics. For example, the activity of the +TIP protein Chb/CLASP is regulated by the tyrosine kinase Abelson during axon guidance at the CNS midline [153], which is also a regulator of the actin filament elongating factor Enabled/VASP [164]. Furthermore, GTPases of the Rho-family, which are major regulators of the actin cytoskeleton (see 'Regulators of actin dynamics in *Drosophila *growth cones' above), also influence a number of microtubule regulating molecules [150,156].

### Regulators of actin-microtubule cross-talk

Cross-talk between microtubules and actin is an essential aspect of regulation of growth cone behaviour (see 'Principal structure and function of growth cones' above), and this process has long been known to be facilitated by microtubule-associated proteins [165]. Such a function may, in part, be carried out by MAPs, as suggested by the observation that MAP2 can bind both microtubules and actin [166]. In *Drosophila*, two molecules with dual actin- and microtubule-binding capabilities have been reported. Short stop/Kakapo is a member of the Spectraplakin family of cytoskeletal linker molecules [167], and its cytoskeletal binding activity is required for axon extension [168]. Another actin-microtubule linker in *Drosophila *is the highly conserved DPod-1, which has been suggested to be required for growth cone guidance rather than axon extension [169].

In conclusion, many (potential) components of the machinery regulating the cytoskeletal dynamics of growth cones have been identified and characterised to differing degrees in *Drosophila*. It is essential to extend the analysis of these factors both at the subcellular level and in combination with genetic strategies, using the various growth cone models available in *Drosophila *to identify precisely how guidance cues are transduced to direct cytoskeletal re-organisation.

## Signalling mechanisms involved in axonal pathfinding in *Drosophila*

### Nature of axon guidance cues

Cytoskeletal regulation is a house-keeping function required in all cells and, thus, a large common set of molecules serving this purpose would be expected to be present in all neurons. In contrast, the signals involved in growth cone guidance and/or the molecular mechanisms that transduce those signals are likely to differ between different types of neurons, as each neuron makes a specific set of pathfinding decisions to reach its synaptic target. It follows that the neuronal cytoskeletal machinery should be responsive to a broad spectrum of axon guidance cues and signalling pathways. Factors that have been shown to guide growing axons include simple structural barriers such as impenetrable tissues or gaps due to wounding [170], electric fields (galvanotropism) [171,172], light (phototropism) [173], or mechanical forces (stereotropism) that can be either applied externally [174,175] or generated via the growth cone's own pulling forces antagonised by adhesive interactions with the environment [176]. However, the best understood form of axon guidance is through external chemical/molecular cues and their receptive machinery within growth cones. Many ligand/receptor pathways involved in axon guidance have been identified [177-179]. Guidance molecules can be either diffusible, or attached to the extracellular matrix or cell surfaces. They are present in the environment of growth cones in an evenly distributed, graded or spatially restricted manner. Depending on its receptive machinery, a growth cone may either fail to respond to certain guidance molecules, or interpret them as growth-permissive, attractive or repulsive signals [2]. It is now a universally accepted idea that the same guidance molecules can act both as repellents and attractants on different axons, as was shown for ligands such as Unc-6/Netrin or Semaphorins in invertebrates and vertebrates alike [180-183]. To add to this complexity, the behaviour of an individual growth cone can be modified over time, for example, through alterations in its electrical activity patterns [184,185].

In the following, we will focus on a subset of neuronal model systems studied in *Drosophila *to illustrate the complexity of the molecular signalling pathways discovered and analysed in this model organism.

## Pathfinding in the trunk of the *Drosophila *embryo

### Axons of motorneurons and sensory neurons

**Figure 5:**
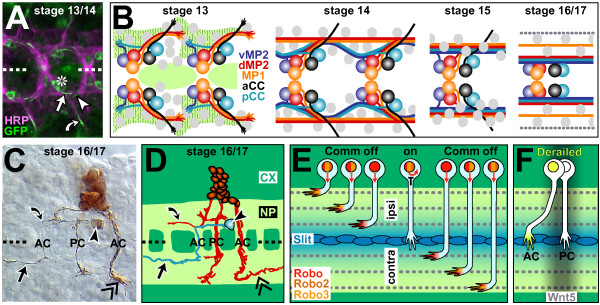
Axonal pathfinding and fasciculation behaviour in the embryonic ventral nerve cord. **(a) **In the ventral nerve cord of stage 13/14 embryos, growth cones of identified neurons (aCC, curved arrow; pCC, arrow; RP2, arrow head) navigate in stereotypic positions (stippled line, midline; asterisk, somata of aCC and pCC). **(b) **Schematic representations of early growing neurons vMP2, dMP2, MP1, aCC and pCC (colour coded): at stage 13, their axons are partly guided by glia cells (grey circles) and Netrin A and B (light green) bound to lateral fields of Frazzled expression (wave pattern); MP1s and dMP2s grow jointly posteriorward, whereas vMP2s and pCCs fasciculate and grow together anteriorward until all four neurons contact one another midway between adjacent neuromeres and establish a single longitudinal fascicle (stage 13) that splits (stage 14), re-fasciculates (stage 15) and splits again (stage 16), partly mediated by glia cells (pCCs, dMP2s, vMP2s form a common fascicle close to the midline, MP1s a distinct axon tract further lateral; grey stippled line represents the lateral Fas2 fascicle of unknown identity; compare Figure 4, ix). **(c,d) **The neuroblast lineage NB1-2 [190] illustrates the stereotypic pathfinding choices of individual neurons (curved arrow, ipsilateral longitudinal path; AC/PC, anterior/posterior commissure; arrow, medial contralateral longitudinal; double chevron, lateral contralateral longitudinal; arrow head, soma of identified TB neuron; CX, cortex; NP, neuropile). **(e) **Regulation of midline crossing and mediolateral longitudinal path choice: ipsilateral neurons don't express Commissureless (Comm), and their combinatorial Robo receptor code determines the mediolateral positioning of their axons; contralateral neurons express Comm (black T), thus preventing transport of Robo receptors to the growth cone (curved red arrow); subsequent downregulation of Comm activity permits the Robo-mediated fascicle choice. **(f) **Choice of anterior versus posterior commissure during midline crossing is partly determined by posterior expression of Wnt5 (Figure 4, xx), which repels growth cones of Derailed expressing neurons. (c,d) Kindly provided by Janina Seibert, Christoph Rickert and Gerd Technau; (b) redrawn from Hidalgo and Booth [223].

A variety of neuronal model systems have been used for studies of axonal growth in *Drosophila *(see 'Models for axonal growth in *Drosophila*' above; Figure 1). Of these, the most intensely studied are the systems in the trunk of the *Drosophila *embryo, in particular, motoraxons in the periphery and axons of motor-, inter-, and sensory neurons within the CNS (neurons 1–3 in Figure 1). From developmental and morphological analyses of these neurons, it has become clear that the identity of individual neurons is specified mainly by their stereotypic lineages [186-188]. Each neuron then makes a number of stereotypic pathfinding decisions as they advance to their particular target and the mature morphology of many of these axons have been characterised (Figure 5c,d) [189-193].

For example, motorneuronal growth cones make a series of choices as they grow into the periphery. First, they have to take the appropriate stepwise decisions that guarantee that their axons exit the CNS and join one of six possible principal nerve branches leading to their appropriate target muscle fields. Second, they defasciculate from other axons in their nerve at precise points to approach their appropriate target muscles. Third, they establish adhesive contacts with their appropriate target muscles at precise positions. Fourth, the motorneuronal growth cones eventually undergo drastic morphogenetic changes and differentiate into neuromuscular junctions. In this cellular system, we currently have insights into: the specification of motorneuron and muscle identities (which in turn determine motor axonal pathfinding behaviour); relevant cells and tissues used as guideposts by motor neurons; mechanisms of fasciculation, defasciculation and pioneer guidance; and the combination of permissive, attractive or repulsive molecular guidance cues governing motor axonal navigation. The systems utilised for studying motor axon guidance and the mechanisms they have revealed are discussed in detail elsewhere [49,194].

In contrast to the motoraxonal system, the sensory system of the trunk has been somewhat neglected, in spite of its obvious advantages, which include the following. First, each of the approximately 40 sensory neurons per hemisegment has been individually identified [195]. Second, sensory cell bodies are relatively accessible and can be visualised at the single cell level in the living embryo [192]. Third, the normal pattern of axon growth from several of these neurons, and the environment through which they navigate in the periphery and where they terminate in the CNS, have been characterised in detail [192,196-201], providing an ideal background for studies of the genetic control of sensory axon growth. While at least two morphological screens have been performed to uncover such genes [25,30], our understanding of the molecular basis for sensory axon guidance is still limited. Nonetheless, several factors that guide motor axons in the periphery and interneuronal axons in the CNS have been shown to play a role in sensory axon guidance, such as Delta and its receptor Notch [197], the actin-microtubule linker molecule Short stop/Kakapo [25,202], Slit and its cognate receptors Robo and Robo2 [203], and the secreted Semaphorin, Sema2a and its receptors Plexin B and Plexin A [204].

### Principal axonal pathfinding decisions in the CNS

Motor-, sensory and interneurons all extend axons within the CNS. To study their growth decisions in the embryonic CNS, a number of visualisation strategies have been used, comprising global neuropile markers (Figure 4, i) [23], molecular markers for reproducible axon fascicles, such as Fasciclin 2 (Figure 4, ix) [205,206], transplantation-based analyses of neural lineages (Figure 5c,d) [207], labelling of identified neurons through dye injections or via genetically targeted markers (Figure 4, xxi-xxxi) [81,208], or ultrastructural analyses [8]. The level of precision provided by each of these techniques varies significantly, which can make it difficult to compare studies obtained via different strategies. However, when combining the various approaches, a very precise view of neuronal growth processes in the embryonic *Drosophila *ventral nerve cord has been obtained, as outlined below.

One obvious feature of axonal growth behaviour in the CNS is that projections of motor-, inter- and sensory neurons aggregate into a cell body-free region, the neuropile, in which synaptic contacts will be established (Figure 2a). This behaviour of neurites to segregate away from cell bodies and constitute the neuropile seems to be driven by axo-axonal affinity, as illustrated by axons of larger groups of neurons in primary cell culture, which always associate into small neuropile-like arrangements [44]. The neuropile stereotypically forms in the dorsal plane of the ventral nerve cord (Figures 1b and 2a), where many of the pioneer motor- or interneurons that initiate formation of the neuropile are located. In contrast, motor- and interneurons in ventral or lateral positions of the nerve cord, as well as sensory neurons entering through the segmental nerves, have to grow through the cortex towards the neuropile. On their way towards the neuropile, axons of neurons derived from the same neuroblast lineage tend to fasciculate [189,190], a property that is also true for the larval nerve cord [209].

Within the (prospective) neuropile, growth cones are faced with a number of decisions (Figures 1b and 4c,d). They may first simply terminate shortly after entering the ipsilateral neuropile (some interneurons); second, grow towards a peripheral nerve root to exit the neuropile (ipsilaterally projecting motorneurons); third, turn anteriorly or posteriorly to project along longitudinal fascicles in the connectives (see 'The formation of longitudinal fascicles in the embryonic ventral nerve cord' below; almost all sensory axons and some interneurons); or fourth, cross the midline (see 'Growth cone guidance at the *Drosophila *CNS midline'; most interneurons and some motorneurons). Those axons that cross the midline are faced with the first and third choices again on the contralateral side. Those axons that do not leave the neuropile terminate their growth in stereotypic positions, presumably at points of future synapse formation [210].

### Motoraxonal growth towards the CNS exit point

The third and fourth growth decisions outlined above (choice of longitudinal fascicles and midline crossing) have been studied extensively during the past two decades, and insights gained from these studies will be reviewed in detail in the sections 'The formation of longitudinal fascicles in the embryonic ventral nerve cord' and 'Growth cone guidance at the *Drosophila *CNS midline' below. In contrast, virtually nothing is known about the mechanisms underlying axonal growth termination in the neuropile. In the context of the navigational capability of motorneuronal growth cones to exit the CNS, the involvement of only two signalling pathways has been suggested so far: the diffusible ligands Netrin A+B and their receptors Unc5 and Frazzled, and the receptor tyrosine kinase Eph and its ligand Ephrin. Targeted expression of Unc5 or of double stranded Eph or Ephrin RNA in interneurons causes their aberrant exit from the CNS (Figure 4, xxx) [211,212]. However, later analyses using *unc5 *and *Eph *loss-of-function mutations failed to detect equivalent axonal phenotypes, calling the original findings into debate [213,214]. At the cellular level, detailed analyses of the contralaterally projecting motorneurons RP1 and RP3 have revealed that their growth cones depend on the presence of the somata of their contralateral homologues, which are required as stepping stones to continue normal pathfinding towards the neuropile exit points. In contrast, ablation studies have revealed that intimate contacts established by these motorneurons with a number of glial guide post cells are not required [81,82,215]. If RP3 motorneurons are prevented from crossing the midline experimentally (axotomy) or genetically (*commissureless *mutant background; see below), their growth cones will carry out the normal growth regime, however, in mirror image fashion on the ipsilateral side, demonstrating that midline crossing is not an essential prerequisite for their capability to exit the CNS [216,217]. Obviously, the RP3 cell body (ipsi- or contralaterally) lies in a strategic position from which the pathfinding cues towards the exit point can be reached. However, under normal conditions, early growth cones of these motorneurons seem first to be attracted exclusively towards the midline, which dominates over all other pathfinding cues.

## The formation of longitudinal fascicles in the embryonic ventral nerve cord

### The formation of longitudinal fascicles

Detailed analyses of the developing ventral nerve cord have revealed the time course of initial antero-posterior axonal growth within the longitudinal connectives [8,82,218,219]. These investigations demonstrated that a small number of early ipsilateral interneurons, namely MP1, dMP2, vMP2 and pCC, pioneer a scaffold of longitudinal fascicles that are subsequently joined by other ipsi- and contralateral axons projecting in anterior or posterior directions (Figure 5a,b). Cell-specific ablations of MP1, dMP2, vMP2 and pCC in various combinations have demonstrated that these pioneer axons are indeed essential for the establishment of connectives as a whole (Figure 4, xxiv) [219,220].

During their initial advance, growth cones of MP1, dMP2, vMP2 and pCC establish intimate contacts with longitudinal/interface glia cells, which line up in the area of the future connectives prior to the axonal growth phase (Figure 5b) [8,206,218,221]. These intimate axono-glial contacts reflect an essential role of this glial population as guidepost cells for the four pioneer neurons, since ablation of these glia cells causes severe disruption of the pioneer neurons' pathfinding and subsequent establishment of connectives (Figure 4, xxxii) [222,223]. Additional guidance cues for early pioneer neuron growth are the secreted factors Netrin A and Netrin B. Both these proteins are released from midline glia cells and become bound in restricted dorso-lateral areas of the ventral nerve cord by neurons in these areas that express Frazzled (a member of the DCC immunoglobulin subfamily; Deleted in colorectal cancer) on their dorsal surfaces (Figure 5b) [224]. Frazzled is able to bind Netrins in these lateral areas, since it is one of two identified Netrin receptors [225]. This distribution of presented Netrins is used as a navigational cue by outgrowing pioneer neurons, as was demonstrated for dMP2 [224]. Thus, Frazzled acts in a non-autonomous fashion in the context of longitudinal pioneer growth. Whether it is also the receptor used by the pioneering growth cones has not been explored [224].

In addition to responding to external cues such as glial surfaces and Netrins, the axons of the four pioneer neurons interact with each other in a stereotypical way, involving sequential steps of fasciculation, defasciculation and refasciculation (details in Figure 5b) [219,223]. Two factors seem essential for this complex fasciculation behaviour. First, defasciculation of axonal tracts depends crucially on longitudinal glial cells [223], potentially through physical intercalation of glial protrusions. Second, CAMs are expressed on longitudinal axons in a dynamic fashion and are believed to be the immediate factors regulating selective fasciculation processes. These CAMs comprise N-Cadherin [226], Neurotactin (bearing a catalytically inactive cholinesterase-like domain) [227,228], the GPI-anchored molecules Fasciclin 1 (composed of four Fasciclin 1 domains) [229], Connectin (containing leucin-rich repeats) [230,231], and the three immunoglobulin family members Neuroglian (orthologue of vertebrate L1), Fasciclin 2 (orthologue of vertebrate N-CAM) and Fasciclin 3 [232-235]. Of these, N-Cadherin is strongly expressed throughout the neuropile and its loss causes aberrations of longitudinal fascicles (Figure 4, xxxiv) [226]. Fasciclin 2 is initially expressed only in the pCC pioneer neuron (and its sibling aCC) [234], but its expression gradually spreads to further neurons, constituting a final number of at least 11 longitudinal Fasciclin 2-positive axon tracts in the mature embryonic ventral nerve cord (Figure 4, ix) [236]. In the absence of Fasciclin 2, the selective fasciculation behaviour of the pioneer neurons is considerably impaired, although this does not have other obvious developmental consequences [8,206]. An explanation for this mild phenotype seems to be redundancy of factors involved in axonal pathfinding, as best exemplified by Neurotactin. Neurotactin acts as a homophilic CAM in cell culture assays, but this interaction requires its heterophilic binding to the secreted immunoglobulin protein Amalgam [237-240]. Both ligand and receptor are widely expressed in the developing nervous system, but their absence causes no serious detectable phenotypes. However, *neurotactin *mutant phenotypes emerge in combination with mutations in other neuronal growth-related genes [241]. Thus, embryos carrying loss-of-function mutations of both *neurotactin *and *neuroglian *display fasciculation defects of dMP2 and MP1 axons and disrupted connectives at high frequency (Figure 4, xxxv) [241]. Further strong *neurotactin *mutant phenotypes were revealed in combination with mutations of genes encoding the receptor tyrosine kinase Derailed, the cytoplasmic tyrosine kinase Abelson, or the CAM-like molecule Kekkon, but not with a number of other tested factors (Ptp69D, Ptp99A, Fasciclins 1–3, Pollux and Neuromusculin) [241,242].

As illustrated by *neurotactin*'s genetic interactions, the range of molecules involved in longitudinal guidance of central axons in *Drosophila *clearly goes beyond adhesion factors, and this is supported by a number of further mutant phenotypes. For example, lack of the transmembrane protein Semaphorin-1A and its receptor PlexinA causes disruption of a lateral Fasciclin 2 tract (Figure 4, xxxiii), whereas lack of PlexinB (the Semaphorin-2A receptor) causes defasciculation of a median Fasciclin 2 tract [243-245]. Selective manipulations of [Ca^2+^]_i_-dependent Calmodulin function in the four pioneer neurons affect both their growth ability and fasciculation behaviour [246]. Embryos carrying mutations in the genes *wnt5 *or *glaikit *(encoding a phospholipase D superfamily protein) show severe disruption of connectives and strong defasciculation phenotypes [247,248]. Also, embryos lacking Kuzbanian, a secreted metalloprotease of the ADAM family, or the two receptor-linked protein tyrosine phosphatases Ptp10D and Ptp69D, show severe aberrations of the neuropile. However, in these latter two cases the aberrations are not caused through aberrant growth or fasciculation behaviours of the pioneer neurons, but rather due to the misguidance of neurons growing at subsequent stages [249,250].

The expression of these various neuronal receptors, adhesion molecules and their associated molecules involved in the neuronal response to environmental cues must be precisely regulated by pioneer neuron-intrinsic gene regulatory programmes. One candidate regulator is the neuronal transcription factor Longitudinals lacking (Lola). In the absence of Lola, longitudinal glia cells form normally, but pCC, vMP2 and MP1 growth cones stall dramatically and connectives fail to form [23,251]. *Lola *encodes a variety of isoforms that are expressed within subsets of neurons and these are likely to regulate the expression of many guidance molecules [252].

As discussed above, all these genetic pathways implicated in longitudinal fascicle formation eventually have to act on the cytoskeletal machinery of the growing neurons (Table 1 and Figure 3). In agreement with this assumption, genetic manipulations of various cytoskeletal regulators cause severe disturbances of the longitudinal tracts, as was demonstrated for Chickadee/Profilin, Abelson, members of the the Arp2/3 or SCAR/WAVE complexes, Futsch/MAP-1B, Rho-family GTPases, such as Cdc42, combinations of Rac1, Rac2 and Mtl, or the Rac activator Trio (Figure 4, xxxvi to xl) [95,101,132,134,163]. In conclusion, a clear temporal pattern of the cellular events and requirements in longitudinal pathway formation has been described at high resolution. A good number of genes, comprising adhesion, signalling or cytoskeletal regulatory factors, have been demonstrated to mediate these precise events. These reveal a significant role for interactions mediated by cell adhesive molecules to direct the neuronal fasciculation decisions necessary during the formation of longitudinal fascicles. These decisions are regulated by additional contact mediated signals and effected by the activation of cytoskeletal regulators.

### Mediolateral patterning of longitudinal fascicles

Individual longitudinally projecting axons are positioned at particular locations within the connectives. This is mediated both by selective fasciculation behaviour and the use of protein gradients that establish positional information within the connectives to dictate where individual axons project. As explained above, longitudinal pioneer axons are initially grouped into one fascicle, but they subsequently separate into distinct fascicles in characteristic dorso-ventral and medio-lateral positions (Figure 5b) [219,223]. This may suggest a gradual increase in distinct positional values in the neuropile of the ventral nerve cord. In agreement with this hypothesis, the expression of the three paralogous Roundabout receptors (Robo, Robo3, Leak/Robo2) is largely overlapping at early stages of axonal growth. However, at later stages, these three receptors take on clearly distinguishable expression domains, that is, Leak/Robo2 becomes restricted to the lateral area, Robo3 spans from lateral to medial, and Robo covers the whole width of the neuropile (Figure 4, ii to iv) [253,254]. This provides a combinatorial code based on the number of these three Robo receptor types expressed by each particular axon, thus dictating their spatial arrangements within the connectives (Figure 5e). Experimental changes of the Robo receptor composition/dosage expressed by single neurons are sufficient to shift the position of their projections in the medio-lateral plane (Figure 4, xxix) [205,254-256]. During normal development, the state of Robo receptor expression of individual neurons appears to be determined by their cell-autonomous programmes. Thus, the expression or non-expression of the fate-determining transcription factor Atonal in chordotonal versus multidendritic sensory neurons differentially regulates their distinct medio-lateral fasciculation behaviours by instructing the expression state of the Robo3 receptor in a cell type-specific manner [255]. Similarly, the Lola transcription factor has a role to regulate the expression of Robo within central neurons [257].

The ligand to all three Robo receptors is the secreted factor Slit, which is released from glia cells in the midline of the ventral nerve cord where it is present at its maximal concentration (Figure 5e) [258]. It is also present, although at much lower levels, within the connectives, and this localisation is partly mediated by the heparan sulfate proteoglycan Syndecan (Figure 4, vi) [259]. Axons expressing a higher dosage or greater number of Robo receptor types are more sensitive to the Slit repellent and are positioned further from the midline (Figure 5e) [253,254]. In addition, Robo function inactivates N-Cadherin-mediated adhesion [260]. Thus, higher Robo activity weakens the adhesive interactions driving fasciculation. This might allow growth cones with high Robo expression to progress further laterally and aid in regulating their mediolateral positioning. Consistent with this hypothesis, *N-cadherin *mutant embryos display aberrations of longitudinal fascicle patterns (Figure 4, xxxiv) and, at the identified cell level (*apterous*-expressing neurons), growth cones alter their medio-lateral pathfinding and longitudinal fasciculation behaviour in stereotypical ways (Figure 4, xxxi) [226]. It remains to be seen whether additional adhesion molecules are regulated similarly through Robo-dependent pathways.

Interestingly, axons shifted in the medio-lateral plane through targeted manipulations of their Robo genes do not project randomly but seem to partition in an organised way with a different group of axons, as suggested by their fasciculated appearance (Figure 4, xxix) [205,254,255]. A potential molecular explanation for this phenomenon is that the types and patterns of adhesion molecules is repeated within different mediolateral positions, allowing shifted axons to find an appropriate fascicle even in an inappropriate Robo zone.

In conclusion, both short-range interactions as well as long range organiser activity contribute to the spatial arrangement of longitudinal fascicles along the medio-lateral axis of connectives.

### Growth cone guidance at the *Drosophila *CNS midline

The majority of axons cross the midline within either of two commissures per segment, called anterior and posterior commissures (Figures 1b, 4c,d and 5i). The formation of these commissures begins at embryonic stage 12 and involves dynamic but reproducible interactions between pioneering growth cones, neuronal cell bodies and migrating glia cells [28], and a dynamic up- and downregulation of cell adhesion molecules, such as Fasciclin 1, during the period of axonal invasion of these commissures [8,261]. Each neuron makes a stereotypic choice as to whether to cross the midline and, for those that cross, whether to grow through the anterior or posterior commissure (Figure 5c,d). Within each commissure axons are arranged in fascicles, as illustrated by the expression pattern of molecules such as the adhesion molecule Connectin, which is restricted to specific sub-fascicles within commissures [230,231].

The Wnt5/Derailed signalling pathway (Figure 5f) plays an important role to regulate growth cone choice of anterior versus posterior commissure. Axons of the anterior commissure express the atypical receptor tyrosine kinase Derailed, which mediates their repulsion from the posterior commissure (Figure 4, xix) [208,262]. A ligand of Derailed that has been shown to mediate this commissure-specific repulsion is Wnt5, which is enriched in the area of the posterior commissure (Figure 4, xx) [248,263]. Absence of Derailed or Wnt5 causes random misrouting of axons from the anterior towards the posterior commissure (Figure 4, xviii), whereas Derailed gain-of-function repels axons from the posterior commissure (Figure 4, xxii and xxvi) [208,248,263]. Obvious candidate factors organising the intrasegmental distribution of guidance cues along the antero-posterior axis are the segment polarity genes. Consistent with this assumption, embryos mutant for the segment polarity gene *gooseberry *have a severely reduced posterior commissure (Figure 4, xvii) [23,264].

With respect to the axonal choice of whether to cross the midline or stay ipsilateral, two major regulatory pathways, the Netrin/Frazzled and Slit/Robo systems, have been identified. Cells at the midline of the ventral nerve cord express the secreted factors Netrin A and B, in the absence of which commissure formation is clearly reduced (Figure 4, vii and xv) [265,266]. Although the Netrin molecules can influence the growth of axons some distance from the midline, this does not appear to be necessary for midline crossing in *Drosophila*, as membrane tethered versions of Netrin that necessarily act over a short-range are sufficient to attract axons across the midline [267]. In contrast, long-range signalling activity of Netrin is necessary for commissural axons to traverse the floor plate of the vertebrate spinal cord [268]. Netrin A and B act as attractive ligands to CNS axons expressing the Frazzled receptor to bring them across the midline (Figure 4, viii and xvi) [225]. However, not all neurons are attracted by Netrins, and neurons expressing the alternative receptor Unc5 are repelled by them (Figure 4, xxx) [212]. First insights are being gained into the spatial regulation of Frazzled expression. Thus, in neurons of the posterior commissure, Frazzled (together with Trio and Enabled) is directly upregulated by the segmentation gene *Engrailed *[269].

Another major factor guiding axons at the CNS midline and opposing the effects of Netrins is the secreted repellent factor Slit, which is also expressed by the glia cells at the midline of the ventral nerve cord (Figure 4, vi) [258,270]. In the absence of *slit *function, all axons of the ventral nerve cord collapse towards the midline (Figure 4, xiv) [23]. Slit activity to control midline crossing is mediated by the two immunoglobulin domain containing receptors, Robo and Leak/Robo2 (see 'Mediolateral patterning of longitudinal fascicles' above). Absence of these two proteins phenocopies the *slit *mutant phenotype (Figure 4, xi) [205,271,272]. Thus, Slit is required to prevent axons from inappropriately reaching the midline; it acts to maintain all ipsilateral growth cones away from the midline, and also ensures that contralateral growth cones are prevented from re-crossing the midline. To avoid a gridlock of advancing growth cones at the midline, the opposing Slit/Robo and Netrin/Frazzled signalling pathways are precisely regulated via a number of mechanisms, which may involve cross-pathway regulation of their antagonising activities [273,274]. Also, the heterotrimeric G-protein AcGq3 has been suggested to assist in setting the balance of attractive versus repulsive cues in growth cones [275]. Alternatively, direct interactions between both signalling pathways may occur, as demonstrated in *Xenopus *where Robo receptors are capable of binding and inhibiting the Netrin receptor DCC [276]. The most crucial regulator of Robo activity during midline crossing in *Drosophila *is the short transmembrane protein Commissureless (Comm) (Figure 4, v and xiii) [23,277]. Comm acts in commissural axons to traffic Robo away from the growth cone surface before and during crossing of the midline (Figure 5e). Subsequently, growth cones are prevented from growing back across the midline by down-regulation of Comm activity within distal regions of commissural axons following midline crossing [207,278]. This mechanism aids likewise in steering contralateral growth cones towards the correct medio-lateral position in the connective (Figure 5e; see 'Mediolateral patterning of longitudinal fascicles' above). The Comm-dependent sorting of Robo away from the plasma membrane has been shown to involve Nedd4 ubiquitin ligase activity [279], although details of this regulation have recently been questioned [280]. Interestingly, no Comm homologue has been found so far in mammals. Instead, midline crossing seems to be regulated by cis-inhibition of Robo1 activity through another Slit ligand, Robo3/Rig-1 [281]. However, it has recently been identified that a Rab guanine nucleotide dissociation factor is required in chick for Robo1 surface expression (E Stoeckli, personal communication), suggesting that activity of vertebrate Robo may, in part, be regulated via intracellular trafficking as in *Drosophila*. Furthermore, interactions between Robo receptors in *Drosophila *may influence each others activity (A Myat, personal communication), suggesting further parallels to the vertebrate system.

*In vivo *observations in normal and mutant *Drosophila *embryos have revealed that growth cones of the ipsilateral motorneuron RP2 form longer and more persistent filopodia in *robo *mutant animals, and that filopodia reaching across the midline have the unusual tendency to persist and develop into contralateral branches [84]. This clearly suggests that Robo influences the cytoskeletal machinery of these growth cones. In agreement with this notion, *robo *interacts genetically with mutations of the actin-plasmamembrane linker molecules beta-Spectrin/Karrussell in the context of midline crossing [282,283] and Robo activity increases activity of the actin regulator, Rac [284]. Furthermore, it was shown that both Enabled and Abelson can bind and genetically interact with Robo, thereby providing a link from Robo to the actin cytoskeleton (Figure 3e) [103,285-287]. This link is further strengthened by the demonstration that Abelson, in the context of midline crossing, interacts genetically with the actin momomer-binding molecule Capulet and the microtubule plus end-binding molecule Chb/Orbit/MAST (Figure 3e) [103,153]. In addition, Abelson links genetically to the Netrin/Frazzled pathway [288]. Thus, Abelson seems to be a major factor orchestrating cytoskeletal dynamics in growth cones crossing the midline. Further components of the cytoskeletal machinery described in the first part of this review (Figure 3, Table 1) have been implicated in midline crossing. These factors include the Rho-like GTPases, Pak, Myosin light chain kinase, the actin-microtubule linkers DPod-1 and Short stop, RhoGAP93B/Vilse, and the RhoGEFs Sos (Son of sevenless), Trio and GEF64C (Figures 3e and 5, xxxvi to xxxviii) [119,132,134,138,143,144,169,284,288-291]. A further 'housekeeping' factor with clear involvement at the midline is the Ca^2+^-binding molecule Calmodulin [289].

Apart from the three ligand receptor systems discussed above, a number of other genes have been implicated in the process of midline crossing and need to be incorporated into any model that seeks to explain how midline crossing is regulated. Thus, Amalgam, a secreted protein with three immunoglobulin domains, and its binding partner Neurotactin, a transmembrane protein with a catalytically inactive cholinesterase domain, interact with Abelson in the context of midline crossing [242]. Another molecule interacting with Abelson in midline crossing is Fasciclin 1, a lipid-linked cell-surface glycoprotein that can act as a homophilic adhesion molecule [261]. Syndecan, a transmembrane heparan sulfate proteo-glycan (HSPG) is expressed on longitudinal and commissural axons, genetically interacts with and physically binds to both Slit and Robo, and promotes their signalling [259,292]. Its function at the midline is partially redundant with the GPI-anchored HSPG Dally-like [259]. Furthermore, Syndecan can bind the receptor tyrosine phosphatase (RPTP) DLAR, present on many axons in the embryonic CNS [293]. Besides DLAR, another three RPTPs are expressed in the *Drosophila *CNS, DPTP69D, DPTP99A, and DPTP10D [250,294]. Of these, DPTP10D and DPTP69D seem to promote Slit/Robo function, and combined loss of both causes a *robo*-like midline-crossing phenotype [250]. Robo signalling is also regulated by Kuzbanian, a member of the ADAM family of metalloproteases, which may be involved in proteolytic activation of the Slit/Roundabout receptor complex [272], while Slit secretion is regulated by Schizo, an ARF6 GEF [295]. The translational inhibitor Krasavietz (Kra) is required for normal midline crossing and *kra *mutations interact genetically with *short stop*, *slit *and *robo *[291]. Also, integrins and their ligands Tiggrin and Laminin A show genetic interaction with Slit in midline crossing [296]. Finally, severe midline crossing defects occur in embryos double-mutant for the genes *kekkon *and *neurotactin*, both of which encode adhesion factors [241]. Whether this phenotype also relates to the Slit/Robo function has not yet been addressed.

Taken together, an ever increasing network of factors has been discovered to play an active role in midline crossing in *Drosophila*, and there is hardly a cellular system of axonal growth for which there is more molecular and genetic information available. Axons must make a decision whether to cross the midline and, those that do cross, must select a commissure, and be shepherded towards, across and then away from the midline. This clearly requires a significant amount of cellular processing for which the principal mechanisms are quite well understood (Figure 5e,f), and work has begun to tie together the various modulatory factors into clear regulatory pathways (Figure 3e). The major ligands Wnt5, Netrin, and Slit mediate the choice of commissure, and growth towards and growth away from the midline. The effectiveness of the different ligands has to be modulated to ensure the growth cones make their appropriate responses. For example, the secretion of Slit is regulated by Schizo, while the action of Slit is modulated by proteoglycans and the promotion of attraction or repulsion at the midline carefully regulated by the integration of multiple downstream effectors to ensure a coherent response (Figure 3e). The activity of the major receptors Robo and Frazzled are finely regulated both transcriptionally and post-transcriptionally in crossing and non-crossing axons to ensure they migrate along their appropriate pathways. Finally, the fidelity of these pathway choices is also influenced by cell adhesive interactions and potentially the activity of receptor phosphatases. A good number of the major players involved in midline crossing are conserved across the animal kingdom [297,298], and this work has already had implications for medical research, as demonstrated by the association of human gaze palsy with progressive scoliosis (HGPPS) with mutations of the *robo3 *receptor gene [299].

## Conclusions and perspectives

A number of well characterised and experimentally amenable cellular systems for the study of axonal growth *in situ *have been established in *Drosophila*. Combined with the genetic tractability of the fruit fly, these cellular systems have helped to uncover an impressive palette of molecular mechanisms underlying axonal growth, many of which can be translated into axonal growth processes in higher organisms. However, a number of challenges and open questions still remain to be addressed in the *Drosophila *system.

First, more analyses need to be carried forward to the identified cell level using cell-specific markers (Figure 4, xv versus xxiii, or xxxi versus xxxiv), thus enabling molecular functions to be assigned more precisely to their immediate cellular role. Also, such analyses would provide better means to study the integration of different molecular pathways or activities, especially when capitalising on the same sets of cells as read-outs. Second, of particular interest in current research on neuronal growth are the mechanisms linking the signalling machinery of axon guidance to the factors regulating the growth cone's cytoskeleton and, thus, its morphogenetic dynamics. To carry out such research in *Drosophila*, work at the subcellular level of growth cones will have to be intensified, and the feasibility of such work has clearly been demonstrated (see 'Growth cones in *Drosophila*' above). Third, with respect to pathfinding mechanisms in the ventral nerve cord, virtually nothing is known about growth regulation in the dorso-ventral axis, which may be due to the fact that the read-outs used in most genetic screens would not easily reveal growth aberrations within the vertical axis. Improved cellular systems and closer attention to this aspect of axon growth should ensure that mechanisms will be found in due course [255]. Future searches for such factors could be facilitated by the fact that whole neuronal subgroups show stereotypic dorso-ventral growth behaviours: most sensory neurons (except for vbd- and dbd-neurons) innervate the ventral neuropile, whereas all motor axons grow towards dorsal neuropile areas, where they form dendrites (Figure 2a) [19,236,300]. Fourth, redundancy of mechanisms is an obvious issue, and more double- or triple-mutant constellations, especially of adhesive and signalling factors, will have to be tested to appreciate their real requirements during nervous system development [241,301]. Fifth, we have still few insights into the transcriptional control over the pathfinding machinery in individual neurons, although a number of transcription factors related to growth behaviour have been described [49,251,252,255,302-304]. Improved means to identify the genes regulated by these factors, such as DamID or ChIP-on-chip technology [302,305], will advance our knowledge at this level of growth control. Finally, a whole palette of mutations with exciting phenotypes has been obtained in various genetic screens (see 'Models for axonal growth in *Drosophila*' above), and many of them are still awaiting their detailed analysis. Consequent work in these directions will ensure that *Drosophila *will continue to contribute essential new insights to the field of neuronal growth.

## Competing interests

The author(s) declare that they have no competing interests.

## Authors' contributions

The first draft of this review was written by NSS together with AP, which was then refined, corrected and complemented by GT and PW. All figures were composed by AP with image contributions by NSS (Figures 3a,b and 4a) and design of the genetic interaction scheme (Figure 3e) by GT.
